# Supplementary material for: The interactome of intact mitochondria by cross-linking mass spectrometry provides evidence for coexisting respiratory supercomplexes
Source: Mol Cell Proteomics. 2017 Dec 8;17(2):216–32. doi: 10.1074/mcp.RA117.000470 (PMC5795388; doi:10.1074/mcp.RA117.000470)
Supplement: Supplemental Data [file supp_RA117.000470_133922_0_supp_23978_fzffwf.zip › spectra_annotation/mito_DR_spectra_annotation/423-1-1-1-2-1.pdf]

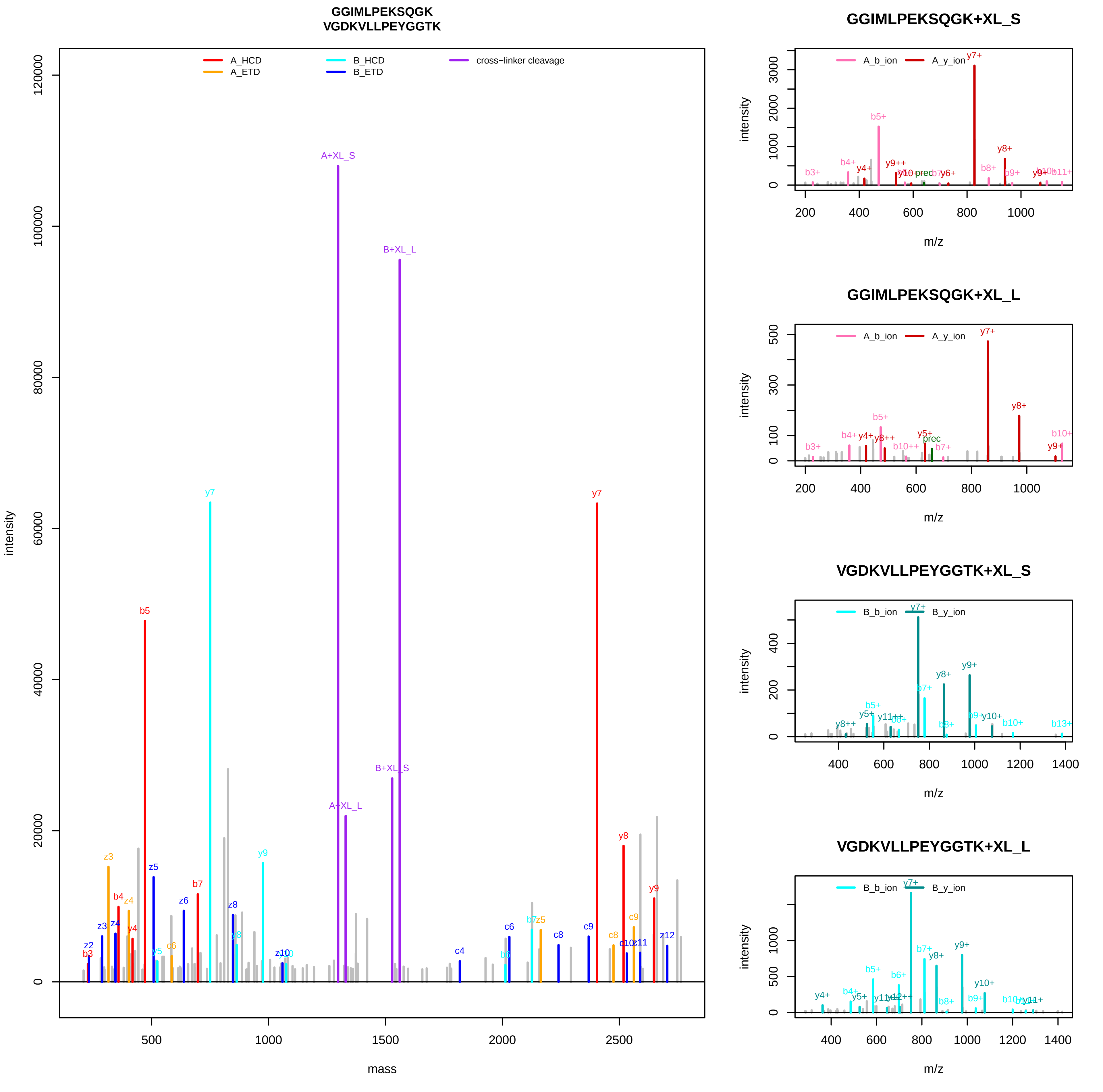
GGIMLPEKSQGK
VGDKVLLPEYGGTK
A_HCD
A_ETD
B_HCD
B_ETD
cross−linker cleavage
intensity
mass
0
20000
40000
60000
80000
100000
120000
500
1000
1500
2000
2500
A+XL_S
B+XL_L
B+XL_S
A+XL_L
y7
b5
y9
z3
z5
z6
b7
z8
b4
z4
y4
z2
b3
y5
c6
z10
c4
c6
b7
z5
c8
c9
y7
y8
y9
c8
c9
c10
z11
z12
GGIMLPEKSQGK+XL_S
GGIMLPEKSQGK+XL_L
VGDKVLLPEYGGTK+XL_S
VGDKVLLPEYGGTK+XL_L
A_b_ion
A_y_ion
B_b_ion
B_y_ion
m/z
prec
y7+
y8+
y9+
y9++
b5+
b4+
y4+
b3+
b8+
b9+
b10+
b11+
y6+
b7+
y10+
y8++
b10++
y5+
b5+
b7+
y11++
b6+
b8+
b9+
b13+
y4+
y11+
y12++
